# Supplementary material for: Anatomical Modeling of Brain Vasculature in Two-Photon Microscopy by Generalizable Deep Learning
Source: BME Front. 2020 Dec 5;2020:8620932. doi: 10.34133/2020/8620932 (PMC10521669; doi:10.34133/2020/8620932)
Supplement: Supplementary Materials — Figure S1: Robustness of the proposed learning scheme to label noise. Figure S2: Graph extraction from the 3D segmentation map. Figure S3: Training and testing data used in our experimentation. Figure S4: Data preprocessing: intensity scaling. Figure S5: Data preprocessing: denoising. Figure S6: Effect of different steps of preprocessing on segmentation quality. Figure S7: Deep neural network architecture. Figure S8: Ablation of TV and preprocessing for segmentation. Figure S9: Effect of total variation (TV) regularization on the segmentation performance. Figure S10: Effect of L2 regularization (weight decay) on the segmentation performance. [file 8620932.f1.pdf]

Short title: Anatomical modeling of brain vasculature by generalizable deep learning

Full title: Anatomical modeling of brain vasculature in two-photon microscopy by generalizable deep learning

Waleed Tahir[1], Sreekanth Kura[2], Jiabei Zhu[1], Xiaojun Cheng[2], Rafat Damseh[4], Fetsum Tadesse[2], Alex Seibel[2], Blaire S. Lee[2,6], Frédéric Lesage[4], Sava Sakadžić[5], David A. Boas[1,2,3], and Lei Tian[1,3,*]

[1]*Department of Electrical and Computer Engineering, Boston University, Boston, MA, USA*
[2]*Department of Biomedical Engineering, Boston University, Boston, MA, USA*
[3]*Neurophotonics Center, Boston University, Boston, MA, USA*
[4]*Biomedical Engineering Institute, École Polytechnique de Montréal, Montréal, QC, Canada*
[5]*Department of Radiology, Massachusetts General Hospital, Harvard Medical School, Charlestown, USA*
[6]*Hacettepe University, Institute of Neurological Sciences and Psychiatry, Ankara, Turkey*
[*]Corresponding author: Lei Tian, leitian@bu.edu

Supplementary material

In this document, we supplement our main manuscript with additional figures and experimental results.

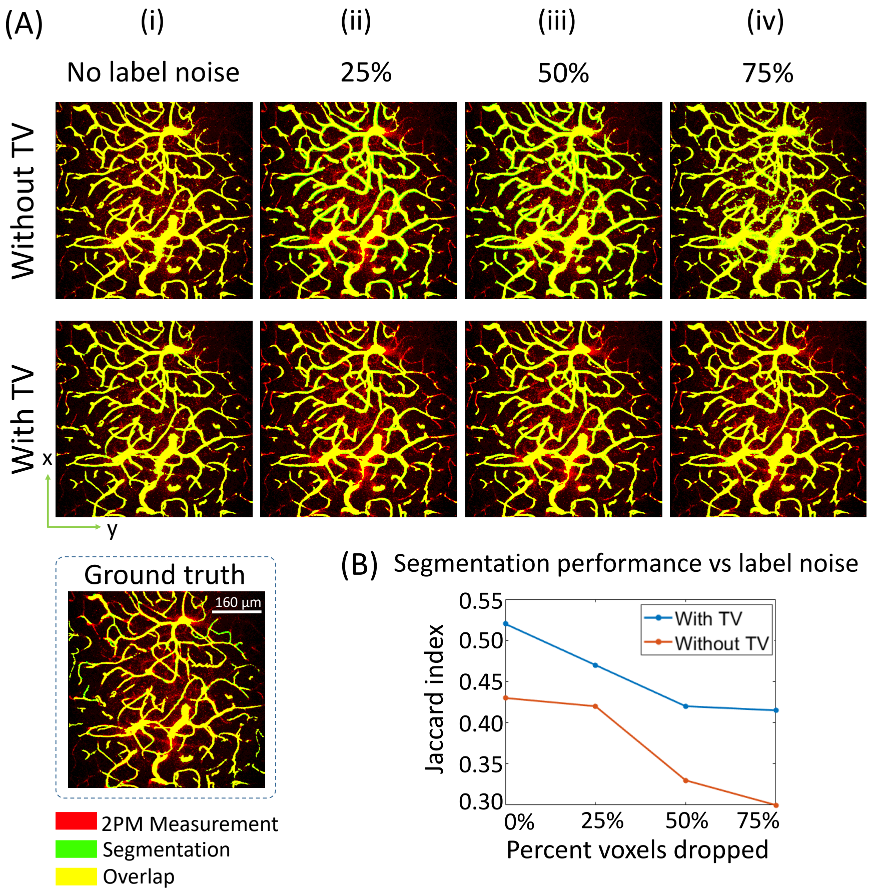

Figure S1: Robustness of the proposed learning scheme to label noise. We train our DNN with various levels of label noise, and demonstrate that adding TV-regularization improves robustness of the segmentation method to label noise. To add label noise, the foreground labels (vessels) were first dilated with a sphere of radius 2. Then a certain percentage of the foreground voxels was selected randomly with uniform probability, and were dropped to zero, such that they now represented background. This method of adding noise was especially chosen to make the edges of vessels ambiguous in the ground truth, as vascular edges are most prone to mislabeling.(A) Qualitative comparison of segmentation performance with various levels of label noise (MIPs $566 - 606\mu$m). The top row represents results from our DNN without TV-regularization ($\alpha = 0$), while the bottom row represents our DNN with TV-regularization. (i) The baseline performance where no label noise has been added to the ground truth. (ii-iv) Label noise is progressively increased from 25% to 75%. The results without TV-regularization deteriorate significantly, while the DNN with TV-regularization is visibly more robust, even in the case of 75% label noise. (B) The quantitative analysis also supports the qualitative comparison between TV and no-TV segmentation; and demonstrates that TV-regularization imparts robustness to label noise.

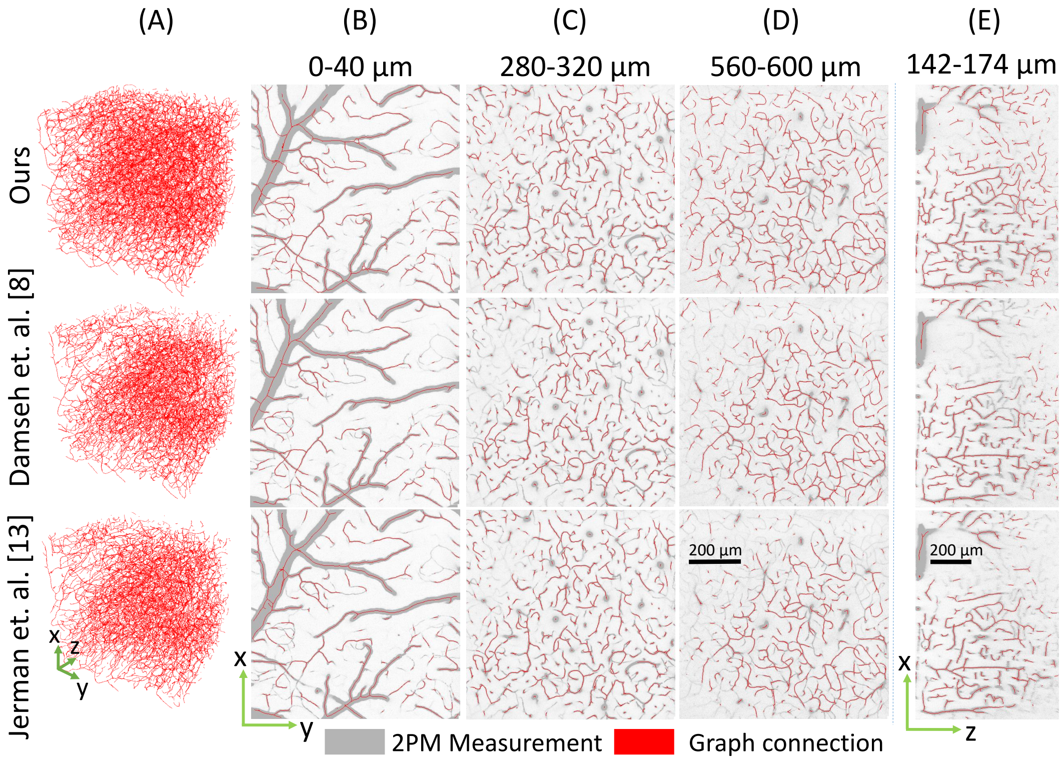

Figure S2: Graph extraction from the 3D segmentation map. The mathematical graph of the vasculature was computed from the segmentation, comprising of nodes connected via edges. (A) 3D view of the graphs, depicted as vascular center lines in the volume. (B-D) MIPs of graphs overlaid on 2PM measurement, each MIP representing 20 discreet slices along z-axis. (E) Longitudinal x-z MIP overlays, each MIP representing 20 discreet slices along y-axis. Graph extraction from our segmentation is qualitatively better compared to other methods, especially below the large pial vessel where measurement contrast is low, and for deep vasculature.

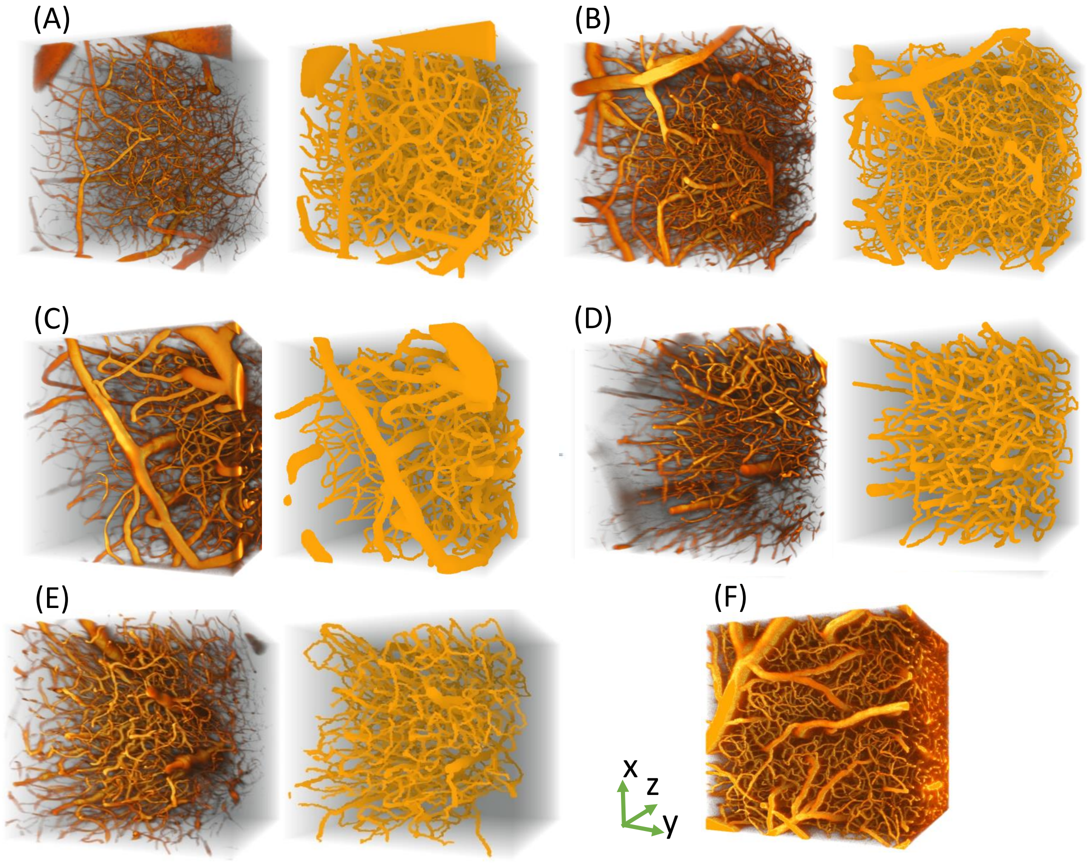

Figure S3: Training and testing data used in our experimentation. (A-E) 2PM measurements and annotated ground truth segmentation pairs for 5 angiograms from setup 1. (F) 2PM measurement from setup 2.

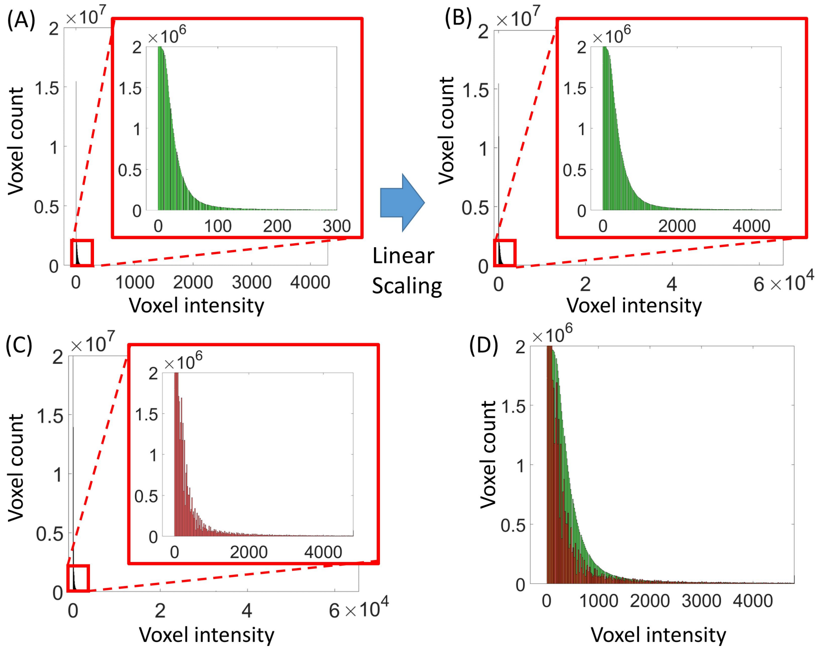

Figure S4: Data pre-processing: intensity scaling. (A) Histogram for the angiogram from setup 2. (B) Histogram in (A) after linear scaling. Scaling is performed on data by multiplying the angiogram with a constant factor to make the intensity scale similar to data from setup 1. (C) Histogram for the test mouse from setup 1. The intensity scale is significantly different from (A) due to difference in bit-depth of camera between setups 1 and 2. (D) Overlay of (B) and (C). The intensity scales between data from setup 1 and 2 become similar after linear scaling on data from setup 2.

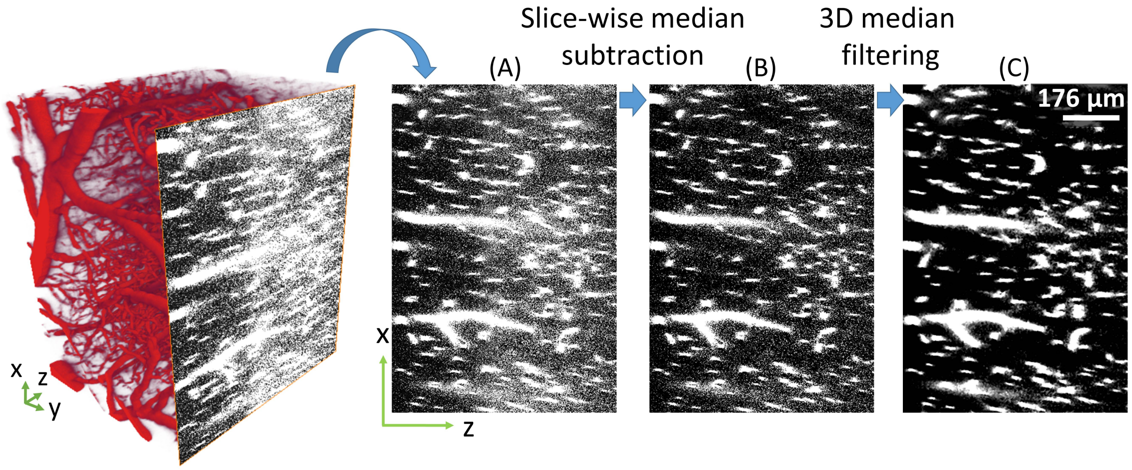

Figure S5: Data pre-processing: denoising. (A) 2PM inherently suffers from signal degradation with imaging depth. (B) Subtracting from each 2D image in the 3D stack, it's median value, visibly improved the quality of the angiogram. (C) 3D median filtering on the angiogram with a $3 \times 3 \times 3$ window significantly reduced background noise.

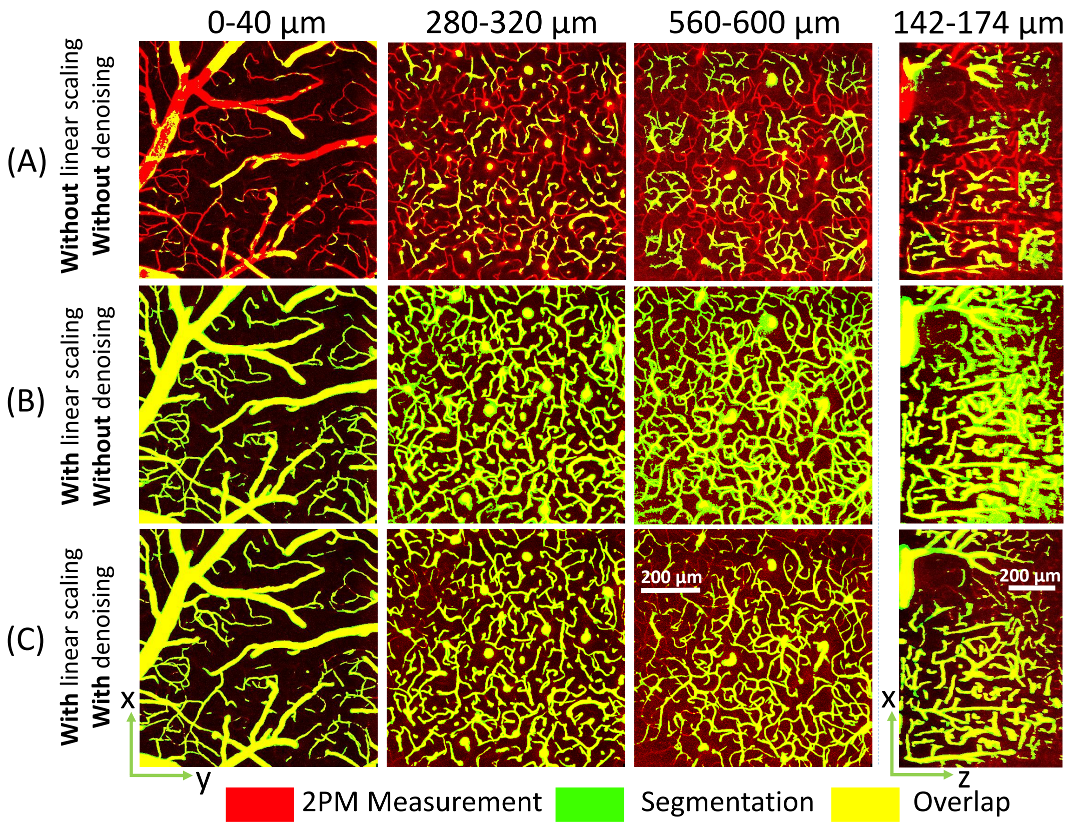

Figure S6: Effect of different steps of preprocessing on segmentation quality. (A) Without any preprocessing on data from setup 2, the segmentation suffers from significant artifacts. (B) Applying linear scaling improves the number of vessels recovered in the segmentation. However, there is a significant number of false positives on vessel boundaries, leading to many adjacent vessels being joined together in the segmentation map. (C) Application of slice-wise median subtraction and 3D median filtering, jointly referred to as 'denoising', further improves the segmentation. Even though the challenging region behind the large pial vessel does contain missed vessels in this case, we do achieve significantly better distinction of vessels in the segmentaiton. Note that in all cases above, we use the DNN trained with the loss described in Eq.(3).

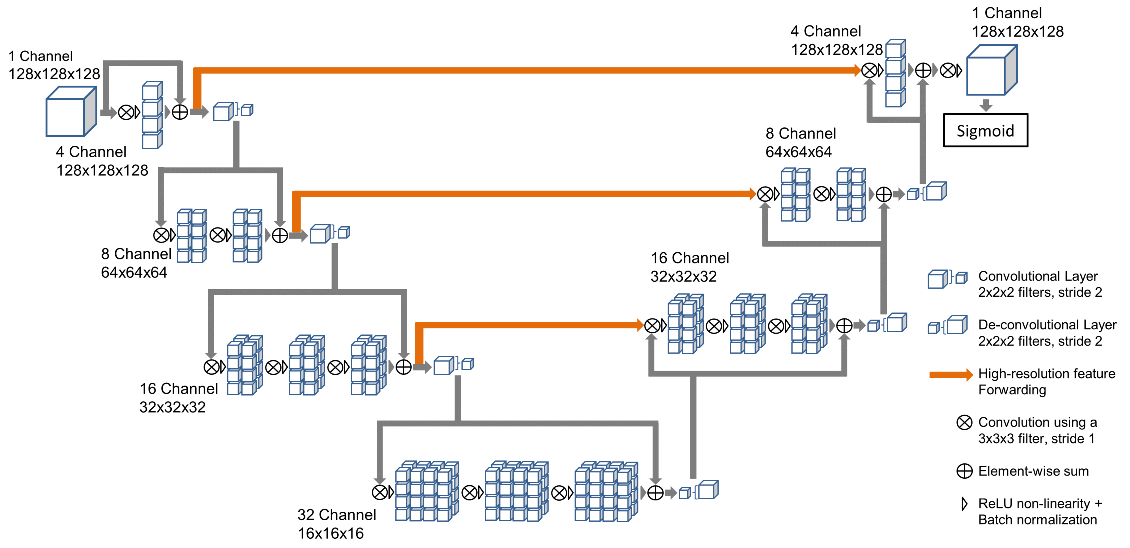

Figure S7: Deep neural network architecture. Our network is designed to be fast and generalizable with good segmentation accuracy. The network is end-to-end 3D taking into account 3D context. Skipped connections forward high-resolution features. Batch normalization improves generalizability and convergence speed, and the number of layers and weights are chosen so as to minimize processing time and over fitting, while maintaining segmentation accuracy.

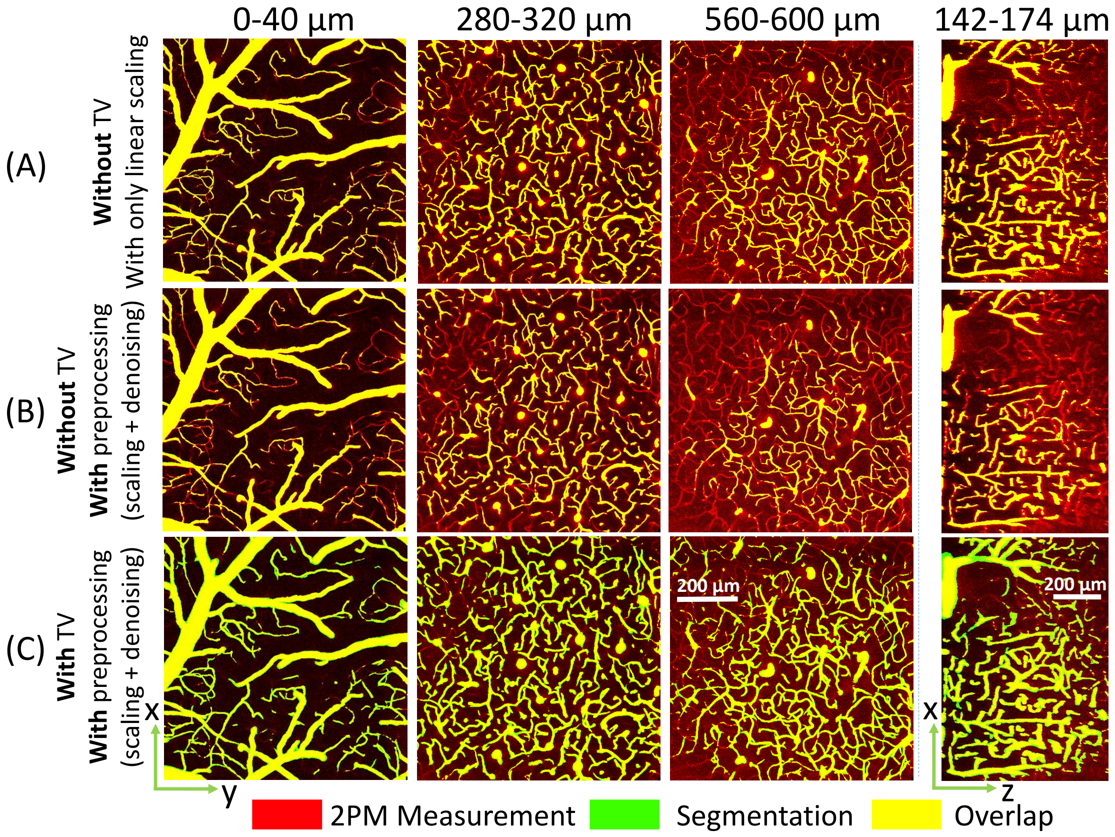

Figure S8: Ablation of TV and preprocessing for segmentation Here we compare the performance of our DNN with and without TV regularization in the loss function, and two levels of preprocessing. (A) Here only linear scaling is performed on the input angiogram before segmentation with a DNN trained without TV. There are a significant number of missed vessels especially in the region below the large pial vessel. (B) Here complete preprocessing is performed on the input angiogram including linear scaling and denoising, before segmentation with a DNN trained without TV. (C) Here TV is added to the DNN loss, in addition to performing preprocessing on the input angiogram, and we see that the segmentation quality is significantly improved. Since this segmentation is on an anigogram from setup 2, it also points towards improved generalization as a result of TV regularization. It is noteworthy that without any preprocessing at all, i.e. no linear scaling and no denoising, the network output without TV regularization was majorly all zero.

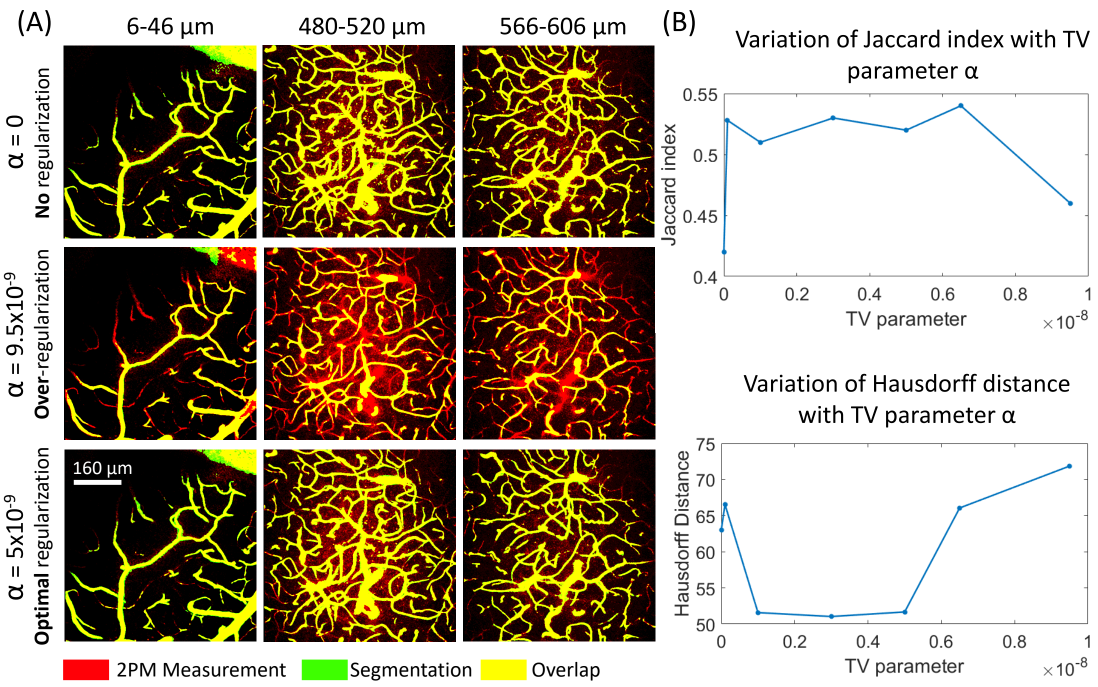

Figure S9: Effect of total variation (TV) regularization on segmentaion performance. (A) Without any regularization ($\alpha = 0$), we observe visible noise in the background, and non-smooth vascular boundaries. When the regularzation parameter is set very high, the segmentation has many missed vessels as the DNN tries to minimize TV. In the optimal TV range, the segmentation quality is the best, both quantitatively and qualitatively. (B) We present the quantitative segmentation quality as a function of the TV parameter α, using Jaccard index, and Hausfdorff distance, as metrics. We find the optimal range of α to be about 1×10^{-9} to 5×10^{-9}.

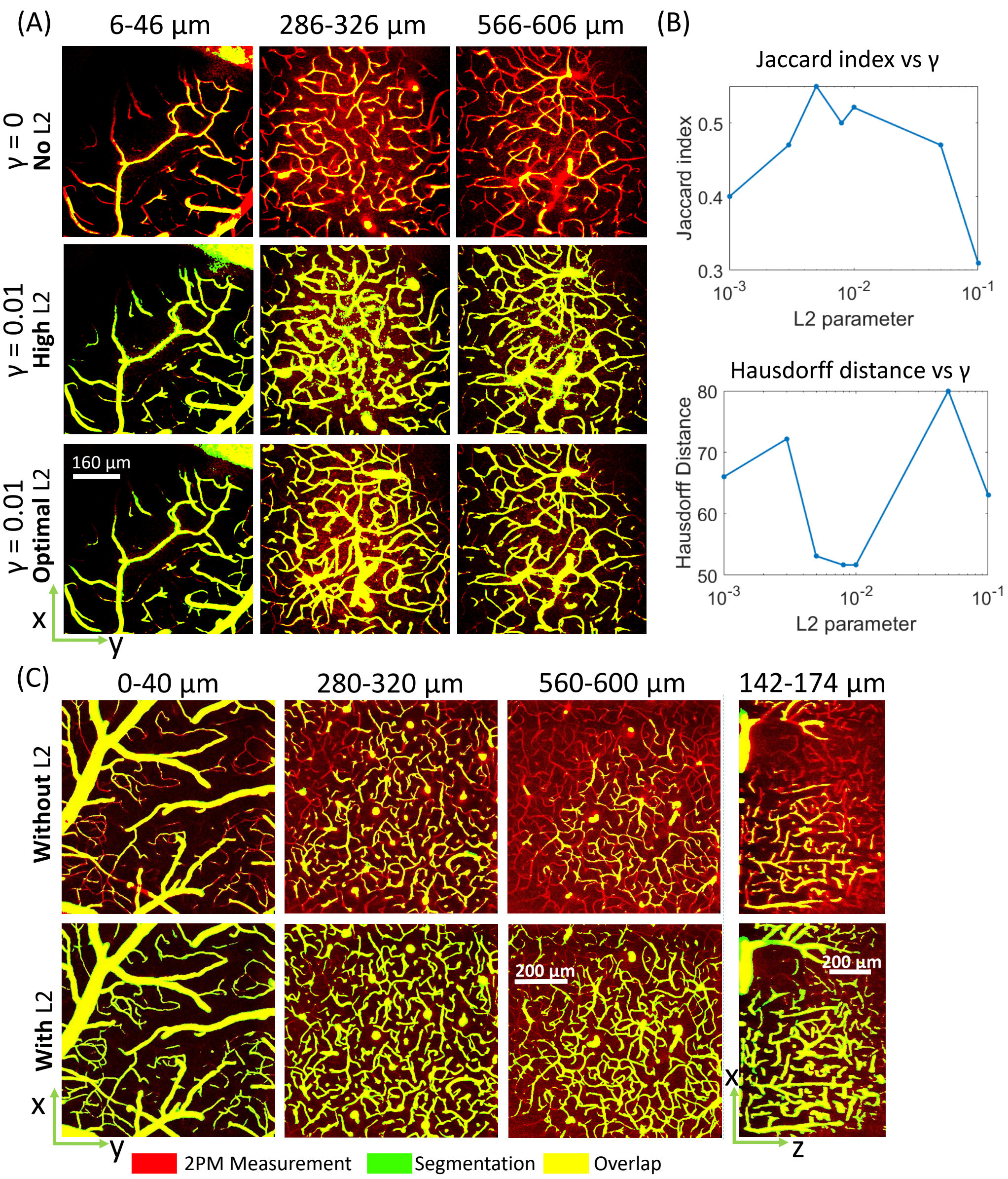

Figure S10: Effect of L2 regularization (weight decay) on segmentaion performance. (A) Without weight decay ($\gamma = 0$), we observe sub-par segmentation performance and a significant number of missed vessels. When γ is set very high, the weights are unable to optimize, resulting in bad segmentation performance. In the optimal range of γ, we obtain best segmentation performance. (B) We present the quantitative segmentation quality as a function of weight decay, using Jaccard index, and Hausfdorff distance, as metrics. We find the optimal range of γ to be about 5×10^{-3} to 1×10^{-2}. (C) Here we compare segmentation maps for the angiogram from setup 2, with and without weight decay. We see that weight decay significantly improves the segmetnation quality, and thus improves the generalization ability of the DNN.
